# Supplementary material for: Bioinspired oxidized mRNA lipid nanoparticles for ex vivo engineering of chimeric antigen receptor macrophages targeting solid tumors
Source: Bioeng Transl Med. 2026 Mar 30;11(3):e70138. doi: 10.1002/btm2.70138 (PMC13247429; doi:10.1002/btm2.70138)
Supplement: Supplementary file 1 — Data S1: Supplementary Information [file BTM2-11-e70138-s001.pdf]

Supplementary Data for
Manuscript Titled:

Bioinspired Oxidized mRNA Lipid Nanoparticles for *Ex Vivo* Chimeric Antigen Receptor Macrophages Targeting Solid Tumors

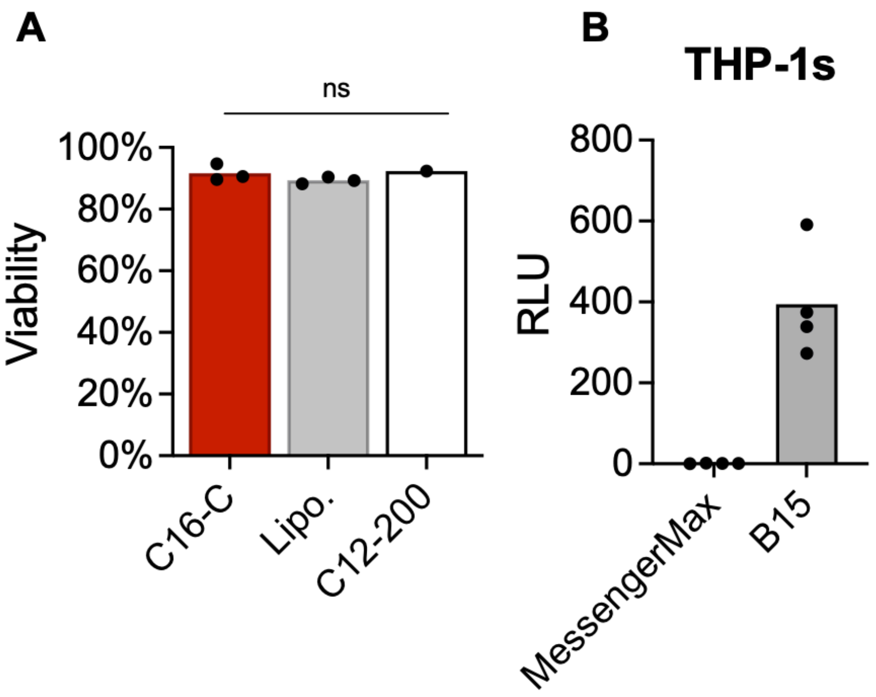

Supplementary Figure 1. (A) Toxicity of the lead C16-C LNP was found to be not significantly different than toxicity associated with other gold standard mRNA delivery vehicles Lipofectamine and C12-200 LNP. (B) PMA-differentiated THP-1 macrophages were treated with luciferase mRNA delivered using B15 LNPs or MessengerMax transfection reagent at a dose of 250 ng/ 50k cells. Luminescence was measured 24 hours later and normalized to untreated controls. Viability was measured using a CellTiter Glo assay in PMA-differentiated THP-1 macrophages after 24 hr incubation and normalized to untreated controls.

Library A

Formulation	C16-C	DOPE	CHOL	PEG Lipid
1	5	4	46	3
2	5	16	31	2
3	5	28	16	1.5
NULL	5	40	61	2.5
4	20	4	31	1.5
5	20	16	46	2.5
6	20	28	61	3
7	20	40	16	2
8	35	4	61	2
9	35	16	16	3
10	35	28	31	2.5
11	35	40	46	1.5
12	50	4	16	2.5
13	50	16	61	1.5
14	50	28	46	2
15	50	40	31	3

Supplementary Table 1. Molar ratios for the Library A formulations screened in the first round of excipient DoE optimization.

Library B				
Formulation	C16-C	DOPE	CHOL	PEG
1	40	4	16	2.5
2	40	8	12	2.5
3	40	12	8	2.5
4	40	16	20	2.5
5	50	4	12	2.5
6	50	8	16	2.5
7	50	12	20	2.5
8	50	16	8	2.5
9	60	4	20	2.5
10	60	8	8	2.5
11	60	12	12	2.5
12	60	16	16	2.5
13	70	4	8	2.5
14	70	8	20	2.5
15	70	12	16	2.5
16	70	16	12	2.5

Supplementary Table 2. Molar ratios for the Library B formulations screened in the second round of excipient DoE optimization.

Formulation	Hydrodynamic Radius (nm)	PDI	mRNA Concentration (ng/μL)
Base	84.15 ± 3.25	0.222	39.40
A1	123.83 ± 1.11	0.205	56.92
A2	102.83 ± 2.92	0.153	43.84
A3	92.40 ± 1.56	0.151	39.92
A4	96.97 ± 3.18	0.151	34.92
A5	94.30 ± 2.30	0.211	30.92
A6	116.60 ± 2.95	0.273	43.56
A7	93 ± 3.33	0.098	33.48
A8	105.78 ± 2.68	0.176	35.76
A9	110.83 ± 4.68	0.276	45.84
A10	92.30 ± 1.81	0.191	33.44
A11	89.43 ± 4.65	0.052	46.24
A12	70.62 ± 2.46	0.170	49.28
A13	78.11 ± 2.14	0.162	26.20
A14	82.37 ± 3.74	0.078	29.20
A15	71.72 ± 5.13	0.159	33.56
B1	85.91 ± 2.13	0.199	40.52
B2	89.30 ± 1.19	0.079	52.34
B3	76.50 ± 4.14	0.284	33.08
B4	70.12 ± 5.91	0.147	41.72
B5	82.56 ± 4.30	0.233	38.60
B6	83.30 ± 2.20	0.194	35.84
B7	77.33 ± 3.59	0.225	36.64
B8	72.68 ± 2.12	0.186	34.42
B9	81.75 ± 4.15	0.178	40.48
B10	83.40 ± 2.46	0.238	48.92
B11	79.26 ± 6.20	0.189	36.92
B12	85.50 ± 3.89	0.259	37.92
B13	75.65 ± 1.44	0.149	30.32
B14	71.92 ± 1.85	0.177	32.32
B15	78.52 ± 2.28	0.129	31.64
B16	87.72 ± 2.75	0.061	41.12

Supplementary Table 3. Physicochemical characterization of LNP formulations tested in DoE libraries A and B. Hydrodynamic radius and PDI were measured using dynamic light scattering and mRNA concentration was measured using a NanoQuant plate.

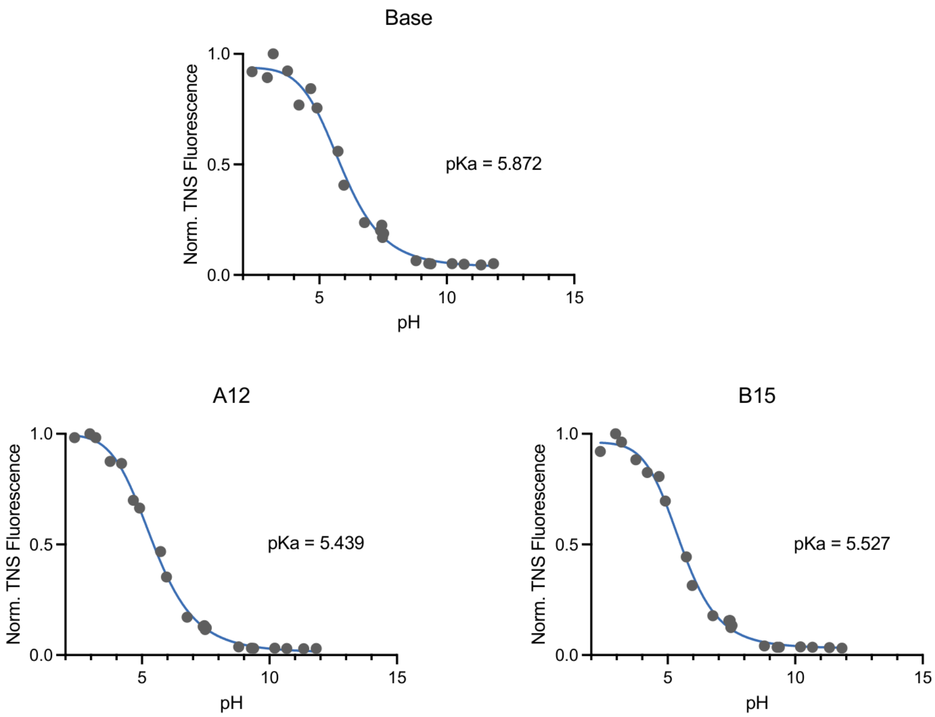

Supplementary Figure 2. TNS fluorescence curves used to calculate the pKa for the Base, A12, and B15 excipient optimized LNPs.

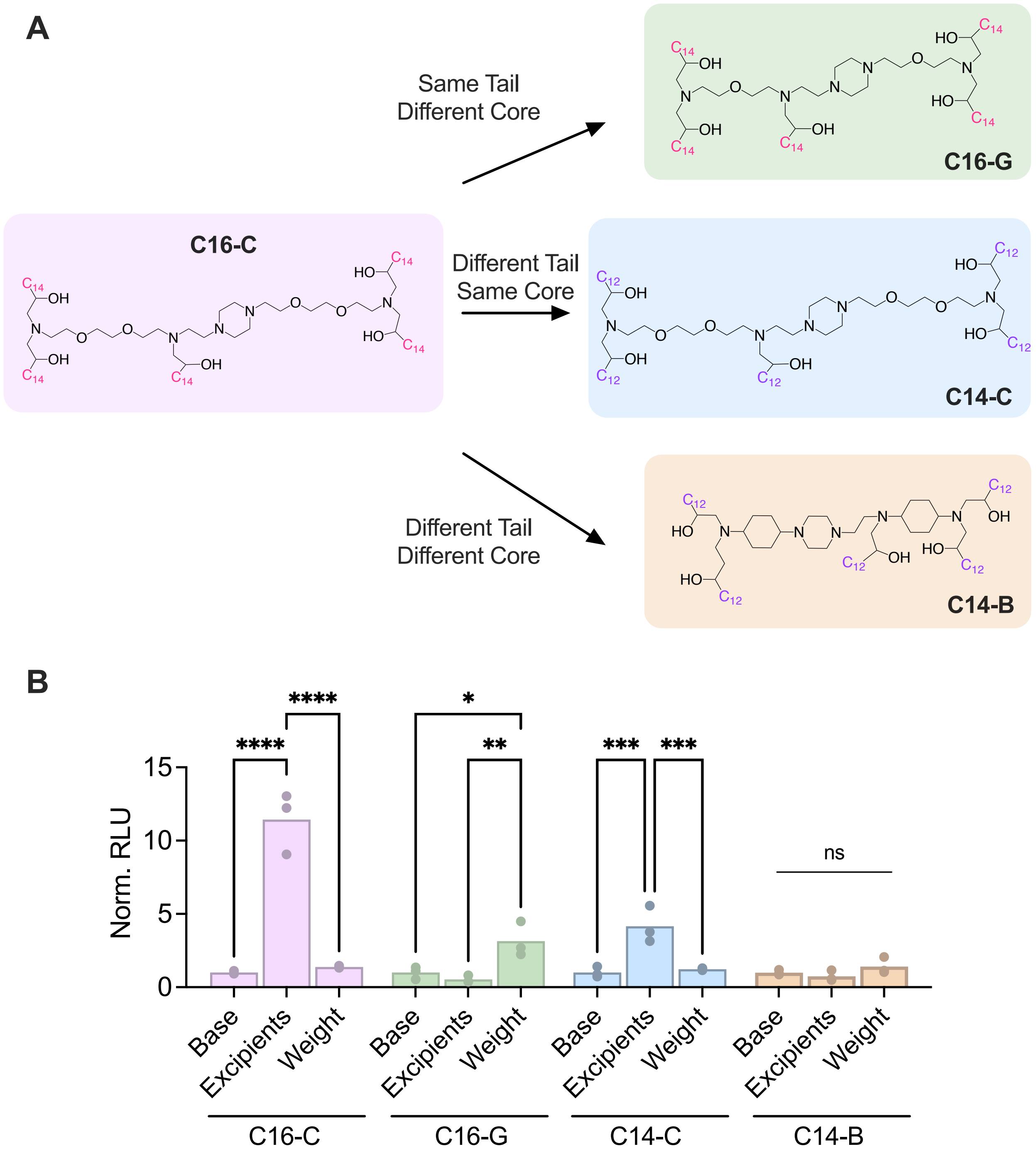

Supplementary Figure 3: Optimization route of LNPs for mRNA delivery to macrophages is unique to each constitutive ionizable lipid. (A) Three additional ionizable lipids from the 24 ionizable lipid library screened in Figure 2 were formulated using the base excipient ratios, B15 excipient ratios or the 17.5:1 ionizable lipid:mRNA (wt:wt) ratio, representative of an excipient-based approach or a weight ratio approach towards increasing the potency of mRNA LNP delivery to macrophages. (B) PMA-differentiated THP-1 macrophages were treated with luciferase mRNA LNPs at a dose of

250 ng/ 50k cells. Luminescence was measured 24 hours later. For each ionizable lipid, luminescence was normalized to the base group and compared using a 2-way ANOVA. $n = 4$ biological replicates. * $p < .05$, ** $p < .01$, *** $p < .005$, **** $p < .001$.

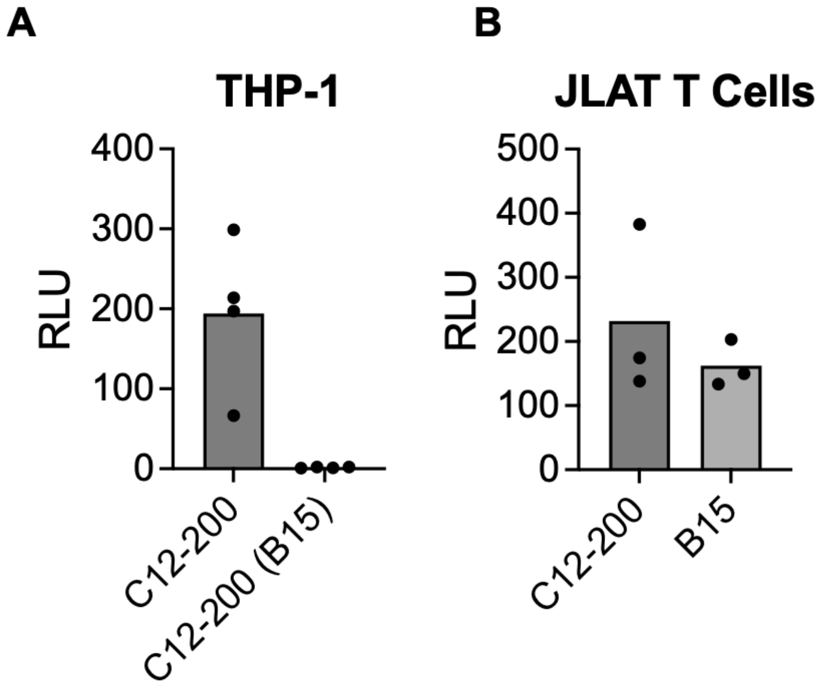

Supplementary Figure 4: Excipient optimization is cell and lipid specific (A) PMA-differentiated THP-1 macrophages were treated with luciferase mRNA LNPs at a dose of 250 ng/ 50k cells. Luminescence was measured 24 hours later and signal was normalized to untreated controls. (B) Human JLAT T cells were treated with luciferase mRNA LNPs at a dose of 60 ng/ 60k cells. Luminescence was measured 24 hours later and signal was normalized to untreated controls.

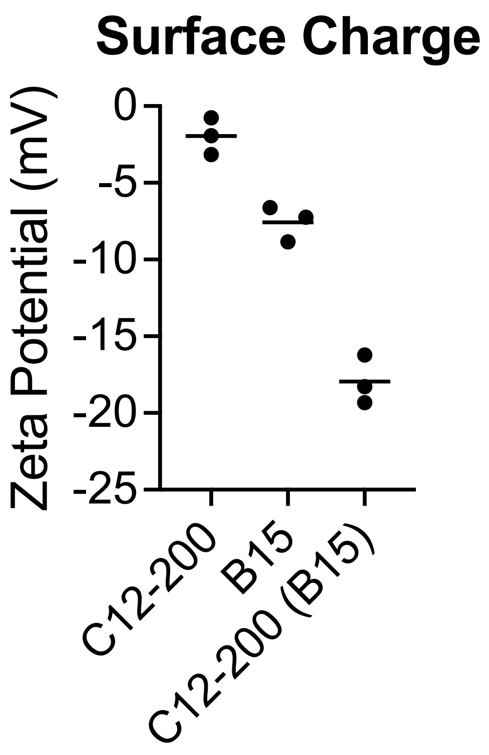

Supplementary Figure 5. Zeta potential measurements of C12-200, B15 and C12-200 LNP formulated with B15 excipient ratios.

ABCG2	CCL19	CD68	EGR2	IL10RB	LXRbeta (NR1H2)	PFKFB1	TIMP-1	B-actin (ACTB)
ACOD1	CCL2	CD80	ENO1	IL12A	Lrg1	PFKFB3	TIMP-2	CYC1
ACTA2	CCL20	CD93 (C1qR1)	EPAS1	IL12p40 (IL-12B)	MARCO	PKM2	TIMP3	EIF2B2
AGGF1	CCL22 (MDC)	CLEC10A	ERG	IL13RA1	MCTP2	PLOD2	TIPE2	GUSB (beta glucuronidase)
ALDOA	CCL24	COL11A1	ETS1	IL13RA2	MERTK	PPARD	TLR2	HPRT1 (hypoxanthine guanine phosphoribosyl transferase)
ALOX15	CCL26	COL1A1	FBP1	IL17	MEST	PPARg	TLR3	TBP
ANG	CCL4	COL3A1	FLT1 (VEGFR1)	IL1B	MMP13	PTGES2	TLR4	
APOBEC 3A.	CCL5	COL5A1	FN1	IL1R	MMP2	RAGE (AGER)	TLR7	
ATG5	CCL8	CREB1	FOXO1	IL4	MMP7	RAMP1	TNF	
Abca9	CCN2 (CTGF)	CTNNB1	FOXO3	IL4Ralpha	MMP8	RIPK3	TNFAIP6	
ApoE	CCR10	CX3CR1	FOXO4	IL6	MMP9	S100a8	TNFRII/CD120b	
Axl	CCR2	CXCL10	FST	IL8	MRC1 (CD206)	SERPINA1	TNFRSF11A (RANK)	
BAX	CCR7	CXCL11	FSTl1	IRF4	MS4A6E	SH3PXD2B	TNFRSF1A	
BGN	CCR8	CXCL12	FYN	IRF5	NAA15	SOCS1	TRAF6	
BTG1	CD11b	CXCL2	GAL3	ITGB1BP1	NFKB1	SOCS3	TYK2	
Bcl2	CD150/SLAM	CXCL3	GATA3	JAK2	NNMT	SPHK1	Trf	
C3AR1	CD16	CXCL9	GLUL	JUN	NOD 1	SPP1	VCAN	
CABLES1	CD163	CXCR2	HIF1A	Jag1	NOD 2	SREBF1	VEGFA	
CACNA1G	CD166	CXCR4	HLA-DRA	Jak1	OLR1	STAT3	VEGFB	
CACNB4	CD1C	CYR61	HSPG2 (Perlecan)	Jak3	PDGFA	STAT6	VEGFC	
CARKL	CD200R1	Cox2	IDO1	KLF4	PDGFB	Stat1	VIM	
CCL1	CD273	DACT1	IDO2	LIGHT	PDGFC	TGFB1	WNT5A	
CCL15	CD274	DCN	IGF1	LIN7A	PDGFRA	TGFB3	cd209	
CCL17	CD36/SR-B3	DNASE1L3	IL-13	LUM	PDGFRB	TGM2	irf1	
CCL18	CD38	EGFL7	IL10	LXRalpha (NRIH3)	PECAM1	TIE1	morc4	

Supplementary Table 4. List of genes studied in donor-derived primary human macrophages via Nanostring gene expression analysis in main text Figure 6.

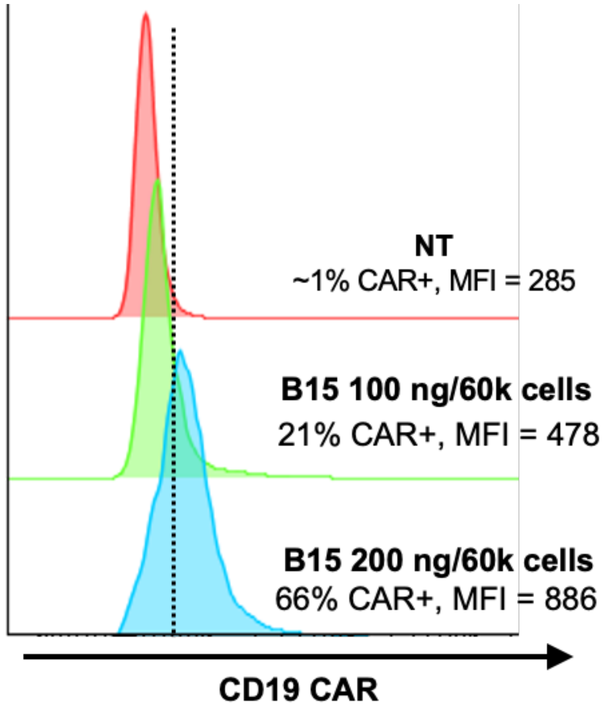

Supplementary Figure 6: CD19-CAR mRNA is expressed more potently than HER2-CAR. CD19-CAR was expressed by 66% of primary macrophages, compared to 18% expression of HER2-CAR at an equivalent dose (Main Text Figure 7B).
